# Development of head-trunk coordination measures for assessing sensorimotor function in laboratory and natural settings using wearable sensors

**DOI:** 10.1038/s41598-025-32201-9

**Published:** 2025-12-18

**Authors:** Hannah M. Weiss, Sarah C. Moudy, Scott J. Wood

**Affiliations:** 1 KBR, Houston, TX USA; 2 Aegis Aerospace, Houston, TX USA; 3 NASA Johnson Space Center, Houston, TX USA

**Keywords:** Head-trunk coordination, Sensorimotor, Adaptation, Algorithm, Engineering, Health care, Neuroscience

## Abstract

Stabilization of the head in space is important for postural and locomotion control. Disruptions in head-trunk coordination can impair functional performance during aging, pathophysiology, or exposure to altered sensory environment states such as spaceflight. Monitoring head-trunk coordination could aid in understanding the risks associated with sensorimotor impairment. The present study evaluated a custom algorithm developed from parameters of head-trunk coordination obtained from wearable sensors. Task performance of healthy adults during standard laboratory tasks both with and without physical restriction via a neck brace were assessed to develop the algorithm and motion thresholds. The algorithm was applied to 12 blinded 4-hr datasets to evaluate the reliability and sensitivity of the measures for identifying altered head-to-trunk coordination when a neck brace was worn during daily activities in a natural setting. The primary head-to-trunk coordination metrics showing high detection accuracy were the root mean square deviation between the angular velocity signals of the head and trunk, followed by the difference in the magnitude orientation. Additionally, the coherence of angular velocity along the X and Z global axes demonstrated good detection sensitivity. The present work lays the foundation for future applications, particularly in monitoring impaired head-trunk coordination in natural settings to provide valuable insights for rehabilitation.

**Supplementary Information:**

The online version contains supplementary material available at 10.1038/s41598-025-32201-9.

## Introduction

Stabilization of the head and neck involves vestibular and proprioceptive reflexes, biomechanical system mechanics and voluntary control^1^. The vestibular-collic and cervico-collic reflexes function, in particular, to dampen mechanical resonance in the vertical plane associated with the gravitational demands during posture and locomotion^2^. It is not surprising that changes in sensory processing during aging, pathophysiology, or exposure to altered environment states such as spaceflight might impair head-trunk coordination. Observations of astronauts recovering from spaceflight provide an interesting context to study alterations in head-trunk coordination due to adaptive changes in both vestibular and musculoskeletal systems^3,4^. A major goal of this research is to develop outcome measures using wearable sensors compatible with natural settings to unobtrusively monitor astronaut’s adaption following g-transitions^5^.

Research following spaceflights has specifically identified alterations in head-trunk coordination involving a decrease in the coherence between head pitch and vertical torso translation during locomotion^6^. The research demonstrated a notable decrease in the primary frequency of head pitch movements, coupled with a sustained forward head tilt during intermittent visual occlusions, attributed to an adaptive strategy to minimize angular head motion^6^. Divergent head movement strategies were identified between multi-time fliers, i.e., astronauts with at least one previous spaceflight exposure, and first-time astronauts. Additional long-duration postflight research also found similar bimodal responses in head and trunk movement latency as estimated by the sinusoidal phase shift between the maxima of the head and trunk body segments for yaw rotational movement during turns^7^. A head-to-trunk locking strategy, or en bloc movement, was identified postflight with seven of thirteen astronauts who had decreased head-to-trunk movement latency. A comparable head-to-trunk locking strategy has been exemplified as an adaptive reaction to systemic challenges caused by conditions such as vestibular impairments^8^ or Parkinson’s disease^9,10^. Previous studies have also shown that children first implement a head-to-trunk stabilization method before transitioning to the more intricate head coordination strategies observed in healthy adults^11^. This voluntary strategic adaptation is suggested to be linked with constraining degrees of movement in cases of conflicting or insufficient vestibular information, aiming to mitigate symptoms like oscillopsia, dizziness, and nausea^6,12^.

In managing posture, the central nervous system must combine head-centered visual and vestibular information with exocentric information from proprioceptive inputs of the lower limbs, trunk, and neck^12,13^. Prior research indicates that discrepancies between the positions of the head and trunk result in changes in the subjective vertical concerning gravitational alignment due to vectorial conflict^13,14^. It is suggested that aligning the head and trunk voluntarily with the gravitational vertical provides a means of stabilizing postural equilibrium by minimizing the necessity to shift between egocentric and exocentric reference frames^13^. Strategies that minimize head movement may additionally support improved processing of the visual and vestibular sensors to control postural equilibrium. During the readaptation to gravitational environments, the modulation of head-trunk coordination may serve as a method for astronauts to restrict head angular movement during locomotion, thereby reducing potential canal-otolith ambiguities^12,15^ to minimize postural instability and motion sickness. While postflight alterations of head movement is evident in previous research, the strategy and extent by which astronauts reduced head motion is under-evaluated across functional tasks beyond locomotion. It is currently unknown whether a consistent head-to-trunk locking strategy is implemented or if an alternative strategy, such as the reduction of head motion in global space, is utilized. While head pitch-to-trunk vertical displacement latency^6^ and head-trunk rotational yaw latency^7^ have been the predominant measures to characterize an astronaut’s head-trunk coordination during locomotion postflight, there is still a knowledge gap concerning the alignment of the head and trunk along dimensions of accelerations, angular velocities, and orientations for the same axis of motion. Moreover, the specific thresholds at which the alignment between the head and torso occurs along these dimensions of movement during standardized functional tasks have been unaddressed in postflight research. Sensitive and accurate measures to assess head-trunk coordination during crew adaption postflight are needed to understand the time course of recovery to baseline performance and provide insight for rehabilitation protocols and crew readiness for the resumption of daily activities.

Various methodologies within existing literature have emerged to quantify the degree of coordination between the head and the trunk among diverse populations. These methods include evaluations of head and trunk amplitude and frequency,^12^ phase difference,^16,17^ and coupling angles^18^ between the head, lumbar, and cervical segments; angular displacement latencies and segmental separation;^19^ cross-correlation;^20^ coherence functions;^17^ rotational speed equivalence;^21^ the area under the curve of rotational amplitudes,^8,15^ and the anchoring index^22–24^. These studies primarily aimed to assess and quantify abnormalities in head and trunk coordination resulting from vestibular decrements and aging or strategies used during specific goal-oriented tasks. At present, there is no prevailing standardized approach for identifying periods when head-to-trunk stabilization strategies are implemented, especially for the determination of coordination across extended data captures where strategies may be variable with respect to the task performed. Directly evaluating the sensitivity of measures for detecting head-to-trunk stabilization would significantly enhance our understanding of adaptive head strategies across diverse groups, encompassing astronauts, individuals with vestibular decrements or pathology, and aging populations.

The objective of this study was to establish and validate an algorithm derived from parameters of head-trunk coordination, specifically designed to detect head-to-trunk stabilization strategies during long duration data captures in natural settings. This framework builds upon existing IMU-based coordination metrics by incorporating laboratory-calibrated thresholds to enable automated detection of head–trunk restriction in natural, non-laboratory environments. A neck brace was utilized for physical restriction of the head with respect to the torso to compare controlled en bloc motion to nominal motion without restriction. By establishing head-to-trunk motion thresholds during known discrete tasks, an algorithm was developed to identify changes in head-trunk coordination. The algorithm was applied to a blinded 4-hr long duration data set to determine the sensitivity and reliability of the head-trunk coordination parameters to identify increased head-to-trunk coordination when a neck brace was worn. While previous studies have measured wearable sensors for long durations, e.g., ^16^, a secondary aim was to evaluate the subjective comfortability and unobtrusiveness of novel inertial measurement unit (IMU) attachments for long duration wear. Quantifying head-to-trunk coordination strategies can offer insights into the readaptation status of crew members, providing valuable information for informing rehabilitation protocols and assessing their preparedness for operational tasks or resuming daily activities that might be affected by vestibular disorientation follow gravity transitions. The research has broader implications to other populations where quantifying increased head-to-trunk coordination over long duration data captures in natural settings may inform pathology-induced or age-related changes in postural control strategies.

## Methods

### Participants

Twelve healthy volunteers (six males, six females; mean age 34 years ± 11 SD, min: 26, max: 63) participated in this study. The cohort had an average height of 1.70 ± 0.07 m. Participant selection was directed through the NASA Test Subject Screening Facility, requiring each participant to pass a Class III flight physical. The participants were informed of the procedures before participating and signed the informed consent approved by the NASA Johnson Space Center Institutional Review Board. Participants reported no musculoskeletal or vestibular impairments at the time of testing.

### Procedure

The experimental protocol comprised two sessions conducted on a single day. The first session, an hour-long testing protocol, was completed at the NASA Johnson Space Center, Houston, TX, USA. Participants performed five functional tasks under three testing conditions: a control condition, a physical head-to-torso restriction condition using a medical-grade commercial-off-the-shelf neck brace (Vista Cervical Collar, Aspen Medical Products, Irvine, CA), and a non-invasive vestibular disorientation condition via galvanic vestibular stimulation. The neck brace featured six adjustable heights to accommodate various anthropometries. In healthy adults, rigid cervical collars reduce flexion/extension by 53.7% ± 7.2%, lateral bending by 34.9% ± 6%, and axial rotation by 59.2% ± 5.3% compared to unbraced conditions^17^. A galvanic vestibular stimulation system (GVS), commonly used as a ground analog for simulating postflight locomotor dysfunction,^18,19^ was implemented to promote alterations in head-trunk coordination due to vestibular disorientation. During initial analysis, significant differences between the control and GVS conditions were limited to a minimal subset of angular velocity changes between the head and trunk. As a result, the GVS data were omitted from the presented results. The current study focuses on the comparison between the control and neck brace conditions. The functional tasks were performed in the same order across conditions, starting with the control condition before proceeding to the neck brace and GVS conditions. The functional tasks, described in detail in the following section, were completed in the following order: walk and turn task, 90° turn and look, 90° turn and acquisition, recovery from fall, and the object translation task.

Participants donned two IMU sensors (Opal, APDM Wearable Technologies Inc., Portland, OR), one on the back of the head and one located on the back of the upper torso, near the tenth thoracic vertebrae. The IMUs were directly time synchronized and aligned within the microsecond using the Motion Studio software. The head IMU was adhered to an attachment mount fastened to the back of a baseball cap. The angle of the IMU was aligned with respect to the gravitational vertical at the start of testing. The torso IMU was secured onto the body using a flexible neoprene fabric strap. Both attachment options were adjustable with Velcro straps and were pilot tested to provide unobtrusive and comfortable monitoring of participant motion for extended durations. Perceptions of comfort of the IMU attachment apparatus were captured throughout both testing sessions (Supplementary Methods Comfort Questionnaire).

Upon completion of the first in-lab session, the participants were sent home while wearing the IMU sensors. The second session comprised 4 h of data capture while the participants resumed their daily activities. At two time points, for a duration of 20 to 30 min each, the participants were instructed to don the neck brace and write down the specific don and doff times. The exact time points were initially blinded to the research team to support verification of the developed head-trunk coordination parameters and identification algorithm. Participants were instructed to refrain from stationary sitting activities particularly during neck brace use and were encouraged to take part in dynamic activities as they were able during the 4-hr testing session.

### Functional tasks

**Fig. 1:**
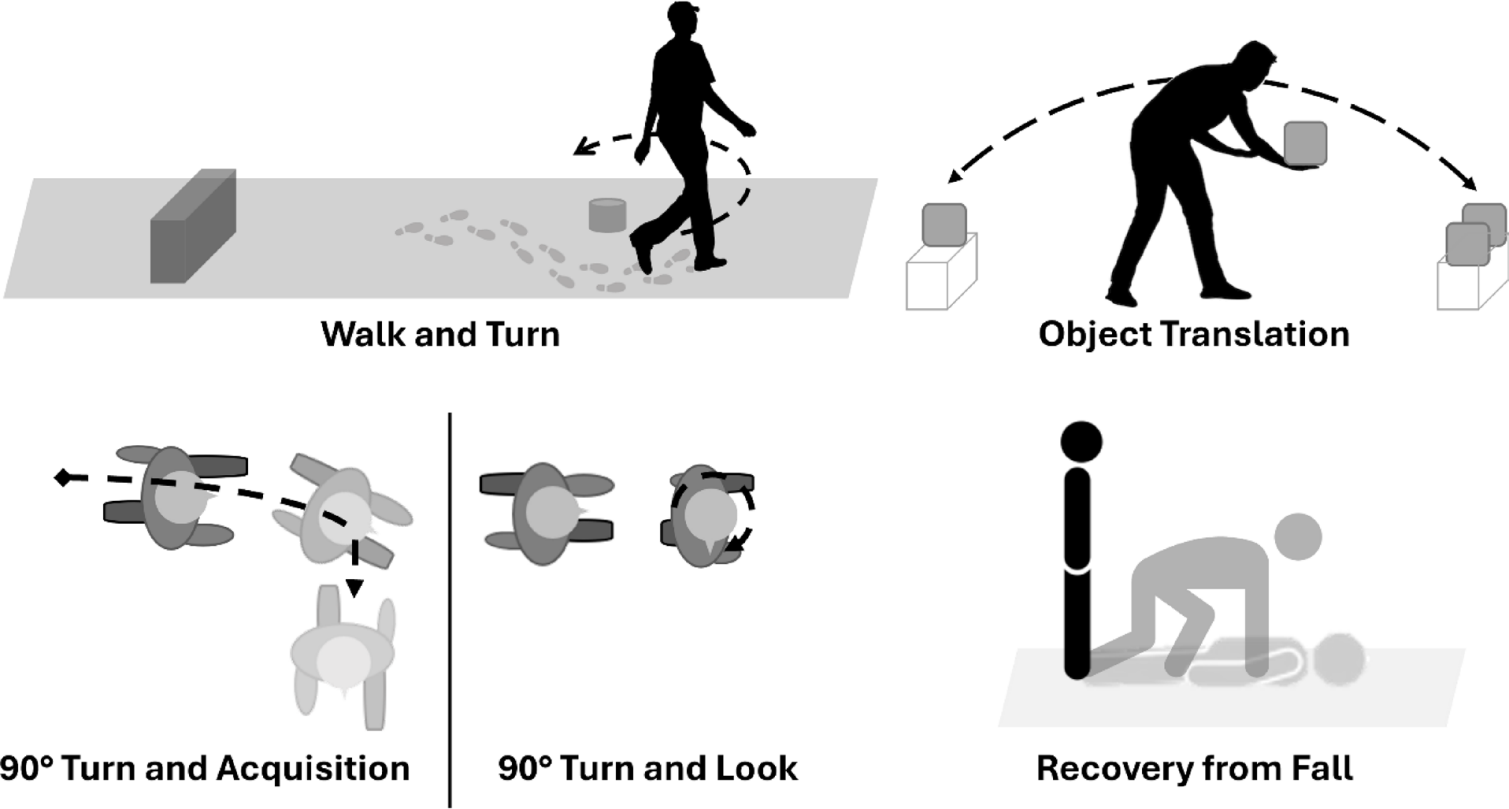
Functional tasks performed during the laboratory session.

The five tasks conducted during the lab session (Fig. 1) included functional tasks implemented postflight for the assessments of astronaut’s sensorimotor performance, known as the Sensorimotor Standard Measures^20^. The selection of these tasks enables direct comparisons to preflight and postflight data of short duration and long duration crew members. These tasks were also selected for their functional attributes that have proven difficult upon returning to Earth from extended exposure to microgravity, namely, head and torso pitch and full-body rotation (i.e., yaw). The chosen tasks involved turning and dynamic movement along all planes of motion to elicit changes in head trunk coordination strategies.

The walk and turn task required participants to rise from a seated position, without using their hands for support, followed by a quiet stance for 10 s. Upon completion of the quiet stance period, participants walked as quickly and safely as possible towards a cone located 4 m away, walked around the cone in a counterclockwise motion, and returned to the chair. On the way to and from the cone, participants stepped over a 30-cm high obstacle. The task concluded once the participant was seated again. The functional demands of the walk and turn task include head and torso pitch, roll, and yaw.

The 90° turn task was a new assessment task designed to examine head and torso coordination during goal-oriented yaw motions for the acquisition of a target. The task was conducted with two distinct variations focused on two separate goals, namely visual target acquisition with a cognitive component and physical acquisition. For both variations, participants were instructed to walk several gait cycles before receiving a random verbal cue, indicating either direction or direction and a number, from the research team. The verbal cues were delivered once the participant had reached a perpendicular distance to two targets located on either side of the ambulation trajectory, requiring a 90° turn of the head and potentially the torso. Each target was a piece of paper placed roughly at eye height, containing three lines of four-character codes comprised of letters and numbers (e.g., K2S4). For the first variation, the 90° turn and look, participants would stop walking and look right or left corresponding to the random verbal cue, visually acquire the target, and recite the corresponding code on the line number given within the verbal cue. For example, a verbal cue of “right, three” instructed the participant to look right and read the code on the third line as quickly as possible. In contrast, the second variation of the task, the 90° turn and acquisition, required participants to dynamically turn right or left after receiving the verbal cue and continue walking along a 90° trajectory until they physically acquired the target with their hand. The functional demands of the 90° turn task was primarily limited to head and torso yaw.

The recovery from fall task was a modified implementation of the traditional task used within postflight research by shortening the duration to only the initial standing portion of the task^20^. In this study, participants were required to lay prone with their hands framing their head before rising to a standing position as quickly as possible. No limitations were applied to the strategy by which participants stood; however, participants were instructed to maintain a quiet stable stance for 3 s. The functional demands of the recovery from fall task primarily included head and torso pitch as well as aspects of roll and yaw, depending on participant strategy.

The object translation task required participants to transfer three weights with handles (2.7 kg, 4.5 kg, 9 kg), one at a time, along a distance of 2.4 m and place them in a receptable 26 cm in height. Participants were able to select the order in which they transferred the weights, divergent from previous protocols. ^20^ The task was completed once all three weights were transferred back to the initial receptacle. Participants were restricted from switching their active carrying hand during a specific transfer to reduce the risk of dropping the weight. However, participants were permitted to complete the task with any strategy they saw fit concerning pitch, roll, and yaw motions.

### Performance measures

**Fig. 2:**
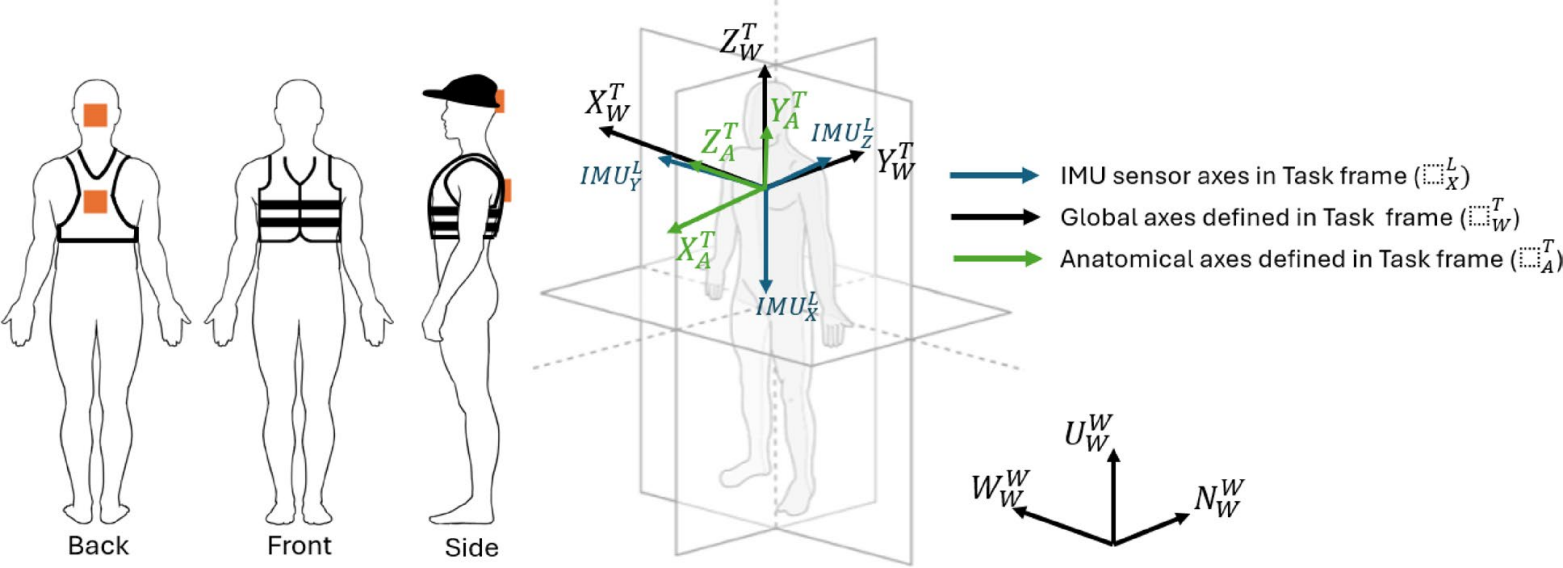
IMU attachments and reference frames.

Head-trunk coordination measures were derived from the IMU sensors located on the head and torso. The use of IMUs enabled the assessment of accelerations, angular velocities, and orientation metrics to inform participants’ postures. The raw accelerations and angular velocities, sampled at 100 Hz, were transferred into a global coordinate system (magnetic north, west, up) and analyzed with a fourth-order low-pass Butterworth filter with a frequency cut-off of 7 Hz. Orientation estimations and sensor-to-segment alignment were performed on the IMU measurements for the head and torso (Fig. 2). A neutral standing position was used as a baseline static posture to calculate the change in pitch, roll, and yaw. For the raw accelerations and angular velocity signals, an extended Kalman filter was used as a sensor fusion algorithm to estimate the orientation of each IMU with respect to the sagittal, coronal, and frontal anatomical planes. The algorithmic process includes defining the local task frame with respect to the IMU sensors, defining the anatomical frames, projecting the relevant global vectors into the anatomical planes,^21^ and computing the orientation angles using the right-handed International Society of Biomechanics (ISB) coordinate system^22^. Positive pitch corresponded to the participant leaning back, positive roll occurred during a rightward lean, and positive yaw was defined when the participant rotated to the left.

Descriptive measures were applied across the accelerations, angular velocities, and orientations for each body segment, namely, the maximum magnitude and the root mean square (RMS) of the signals along each global axis of motion (X - North, Y - West, and Z - Up) across the time series. Comparative measures focused on quantifying relative differences in the signals for the head and torso included the maximum absolute difference, the root mean square deviation (RMSD) (Supplementary Equations, Eq. 1), and the magnitude-squared coherence (Supplementary Equations, Eq. 3). The RMSD compared the overall magnitudes of deviations between two signals. These comparative measures were conducted under the assumptions that a larger magnitude difference of these measurements indicates a lower likelihood of head-to-trunk locking, while a smaller relative magnitude difference may suggest more coordinated movement. The selection of these metrics was based on the understanding that they would be applied to prolonged data recordings, often without comprehensive knowledge of the specific tasks being conducted. Consequently, these measures were designed to evaluate general deviations in the signals rather than focusing on specific functional aspects of the tasks, such as temporal variations in the onset of turn.

### Statistical analysis

Non-parametric analyses using the Wilcoxon signed-rank test were performed for the discrete functional tasks with the control and neck brace conditions. The selected level of significance was 0.05. Effect sizes were calculated using rank-biserial correlation (r).^23^ In the present work, the interpretation of the rank-biserial correlation is as follows: a zero value indicated no relationship, negative values corresponded to a performance outcome that was larger with a neck brace compared to no brace, and positive values suggested larger performance values without a brace compared to the neck brace. The non-parametric analyses were conducted to inform appropriate measures to characterize head-trunk coordination as represented by measures yielding significant differences. The significant measures were applied to the blinded 4-hr datasets to identify time periods of restricted mobility when the neck brace was worn. Performance thresholds were established using the interquartile range (IQR) across the entire dataset of discrete functional tasks, independent of specific tasks. These thresholds were captured to support the identification of neck brace usage, particularly when the task characteristics were unknown during the long duration data capture. Lower bounds of the thresholds were developed from the 25th percentile, while upper thresholds were defined from the 75th percentile.

Following the identification of suitable measures (Fig. 9) and thresholds of performance (Supplementary Table S4), an algorithm was developed to identify regions of head-trunk coordination during the blinded 4-hr dataset. To manage over 48 h of long duration IMU recordings, the data were segmented into 20 min sliding windows, resulting in 12 windows per dataset. Within each window the selected head-trunk coordination measures were evaluated relative to the thresholds established from the discrete tasks in the laboratory setting. Windows in which the data fell within the lower and upper thresholds for neck-brace–restricted motion were first identified. A secondary selection process was then applied to determine the two windows exhibiting the strongest head–trunk coordination by evaluating the lowest relative motion between the head and torso. For coherence measures, the two windows with the highest coherence values within the defined thresholds (Supplementary Table S4) were highlighted. For the maximum and average difference measures, the two windows with the lowest values across the sliding-window analysis were selected. For the long duration data capture, drift adjustments were performed for the IMU orientations by reinitializing the extended Kalman filter in successive time windows, reducing the accumulation of error over extended periods. To further correct slow, gradual deviations, linear drift correction and detrending using MATLAB’s detrend function (The MathWorks, Natick, MA, USA) were applied. These procedures removed low-frequency baseline shifts that can result from sensor bias, slight IMU misalignments, or cumulative integration errors. Yaw wrap-around was preserved to maintain continuity across 360° rotations.

**Fig. 3:**
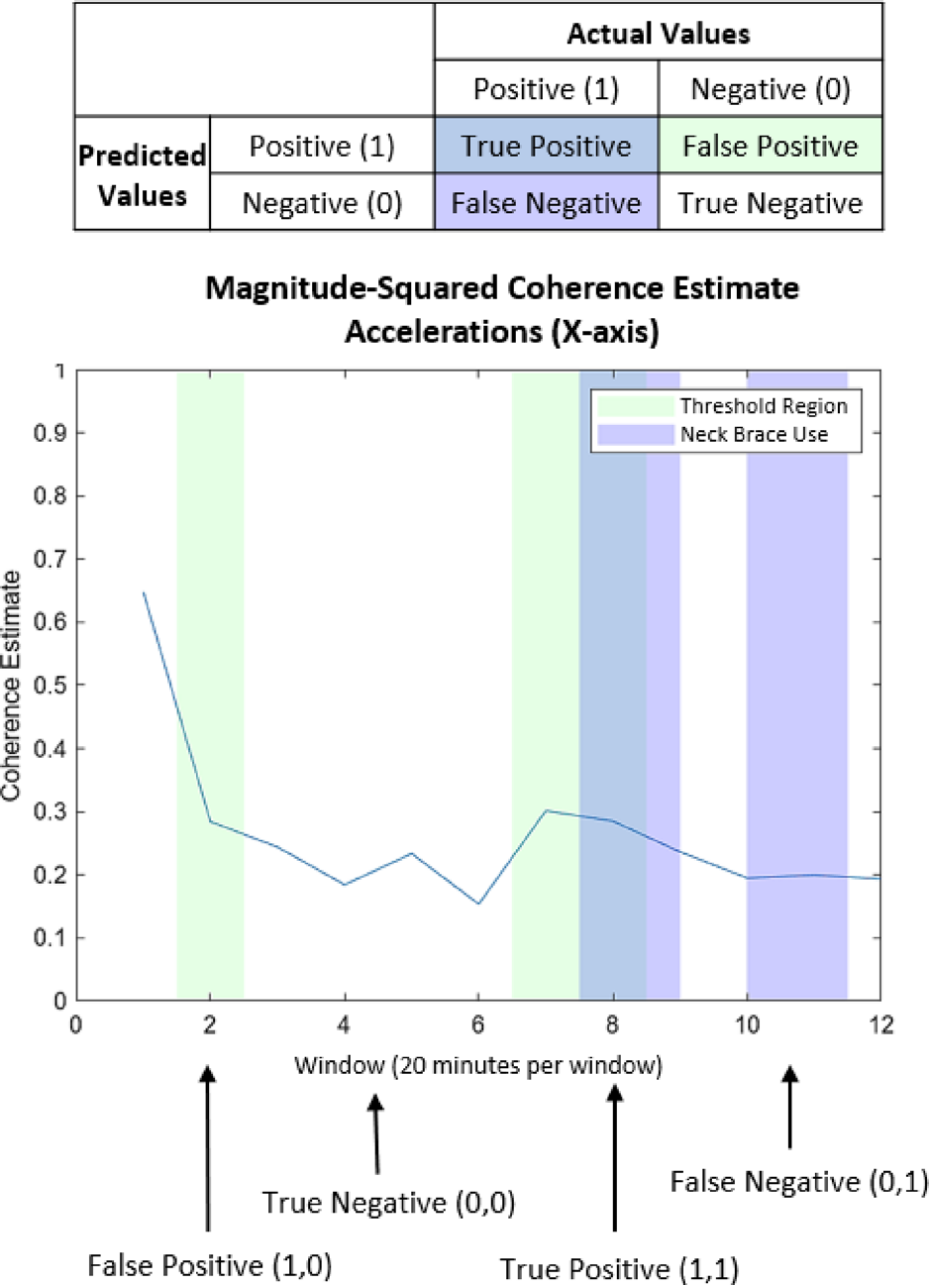
Diagnostic accuracy table. Green shading marks segments in which the algorithm detected increased head-to-trunk coordination; purple shading shows verified neck brace use; blue marks regions where the algorithm correctly identified neck brace use.

Verification of the head-trunk coordination measures was supported through the development of a diagnostic testing accuracy table commonly used in healthcare to determine the appropriateness of diagnostic tools^24^. The diagnostic matrix characterized the accuracy, sensitivity, specificity, precision, classification error rate, and false positive rate for each measure (Fig. 3 and Supplementary Methods Diagnostic Testing Accuracy Formulas). True positives and true negatives were classified as instances where the algorithm correctly identified time periods of neck brace use and no neck brace use, respectively. A false positive was defined as instances when the algorithm identified periods of head-trunk locking when the neck brace was not worn. A false negative was characterized as instances when the algorithm was unable to identify neck brace use when the neck brace was worn. Accuracy is an aggregate measure of classifier performance representing how often the algorithm correctly classified true positives and true negatives across all predictions. Sensitivity, also referred to as the true positive rate, characterized the ability of the algorithm to identify the true positive classifications, while specificity is a measure of how well the classifier identified true negative test results. Precision or positive predicted value represented the portion of positive results that are true positives. The classification error rate represented how often the classifier yielded incorrect predictions across all predictions, while the false positive rate measured the portion of all negatives that yielded positive test outcomes. While all diagnostic measures are reported, the pivotal outcomes for our use case were the accuracy, sensitivity, and precision of the classification algorithm. Given the testing protocol design, false negatives could suggest increased head-to-trunk coordination without the neck brace, which is of secondary concern compared to the true positive rate and the overall classification performance.

## Results

### Discrete functional tasks

The following presents the results and associated p-values for the discrete functional tasks with comparisons to the control and neck brace conditions. Supplementary Table S4 presents the thresholds derived from the 25th and 75th percentiles of neck brace use for the head-to-trunk measures that showed significant effects.

**Fig. 4:**
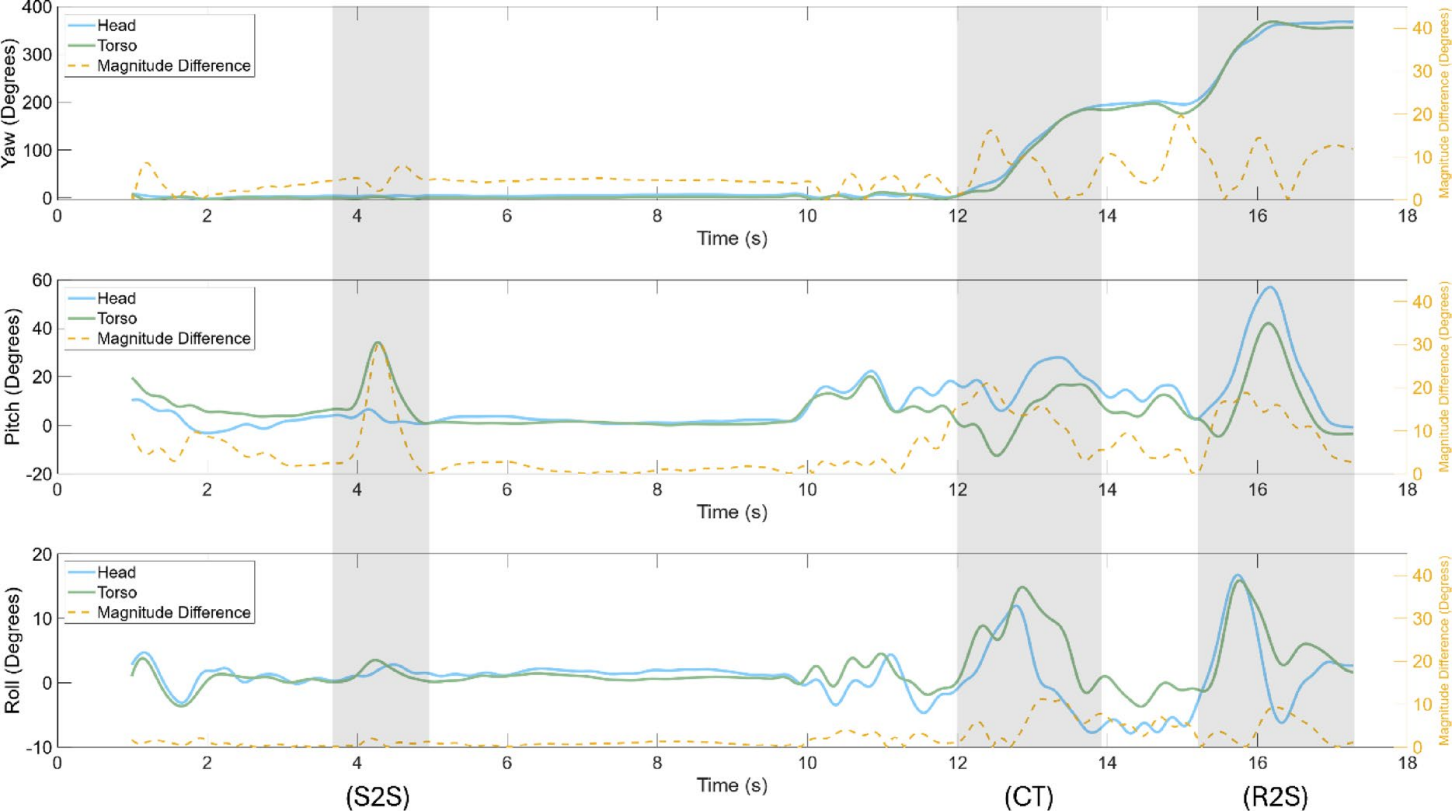
Yaw, pitch, and roll orientation of a single participant during the walk and turn task with the neck brace. The task phases, seated-to-stand (S2S), cone turn (CT), and return-to-seated (R2S), are shaded in grey. Left y-axis: head and trunk orientation (°); right y-axis: head–trunk difference (°).

### Orientation

**Fig. 5:**
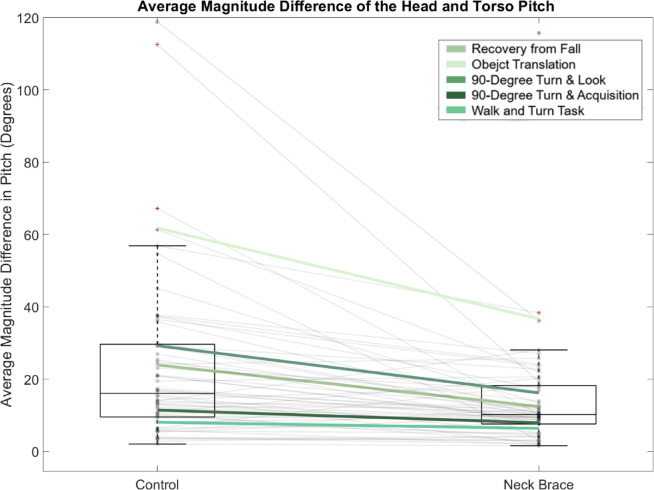
Boxplots representing the task-independent results (i.e., average performance across all tasks) for the RMSD in the pitch plane of motion. The average performance within each task is overlayed corresponding to the key provided.

For tasks with rotational functional demands relative to the vertical, significant decreases in the maximum and average relative yaw difference of the head and trunk were observed in the neck brace condition (Supplementary Table S1) for the walk and turn task (Fig. 4), 90° turn and acquisition, 90° turn and look, and the object translation task. For the tasks requiring functional pitch, the maximum and average relative orientation difference between head and torso pitch was significantly decreased for the neck brace condition while performing the walk and turn task (Fig. 4), the recovery from fall task, and the object translation. The average coherence between the head and torso yaw was significantly increased during neck brace use for the 90° turn and look task, while the average coherence in pitch decreased when compared to the control condition (Supplementary Table S1). No significant differences were observed in the remaining tasks for the coherence of the head and torso pitch motion. The roll plane of motion yielded no significant difference across the dependent measures. Task-independent analyses yielded similar results as the task-specific analyses (Fig. 5), indicating that the coherence and the relative orientation of the head and torso along the yaw and pitch planes identified significant differences and large effect sizes between the control and neck brace use, irrespective of the task.

### Angular velcocity

**Fig. 6:**
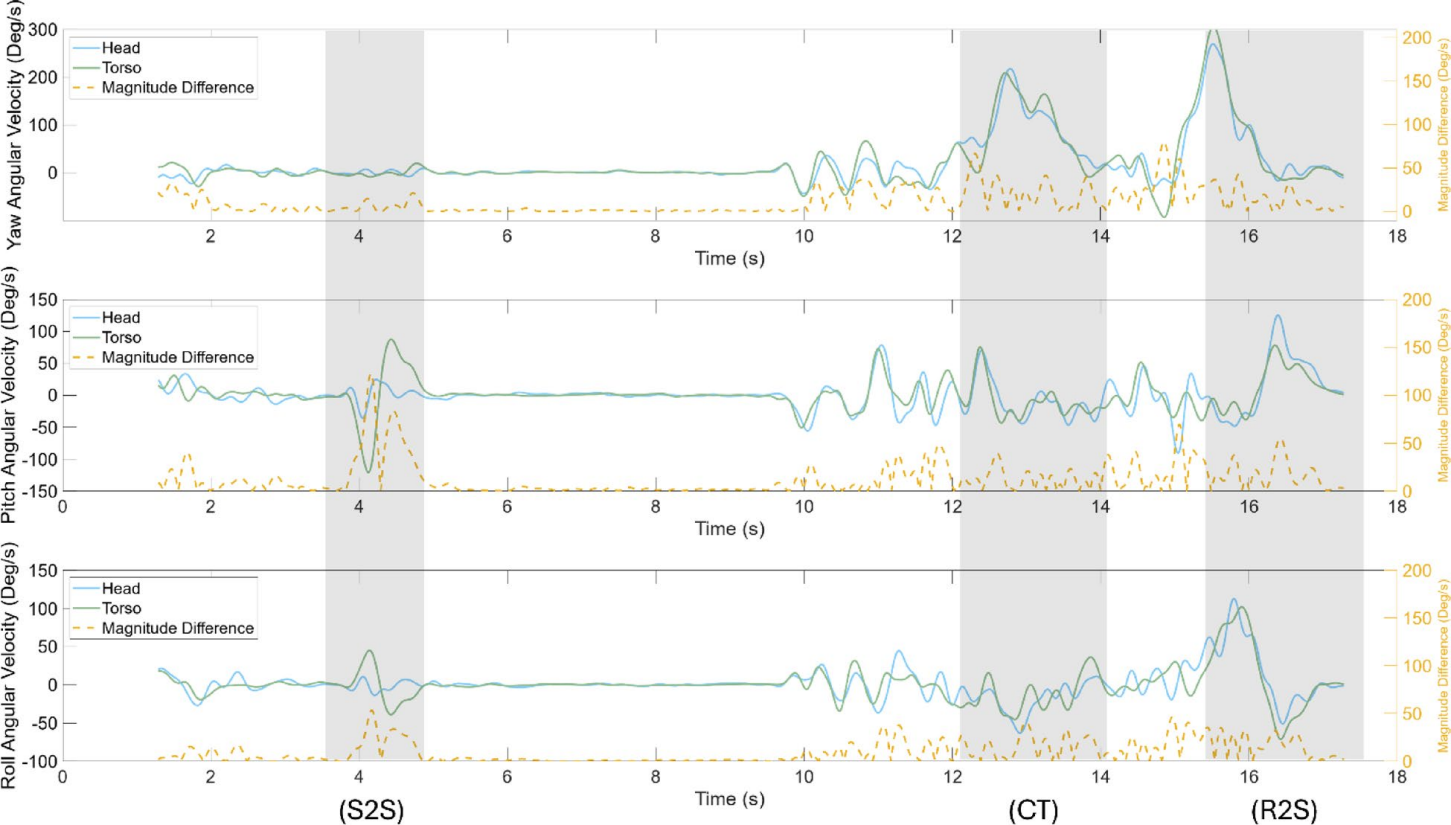
Angular velocity (yaw, pitch, roll) of a single participant during walk and turn with neck brace. The task phases, seated-to-stand (S2S), cone turn (CT), and return-to-seated (R2S), are shaded in grey. Left y-axis: angular velocity (°/s); right y-axis: head–trunk difference (°).

The average relative difference in angular velocity of the head and torso along the global Z axis was decreased significantly with neck brace use for all five tasks, while the average difference along the global X axis decreased significantly for the walk and turn task (Fig. 6), recovery from fall, and the object translation task (Supplementary Table S2). The average coherence between the angular velocity of the head and torso in the X axis increased with neck brace use for the recovery from fall task, while the average coherence along the Z axis increased for the 90° turn and look task. The average coherence of the angular velocity signals of the head and torso along each axis of motion was significantly greater with neck brace use during the object translation task (Supplementary Table S2). Task-independent analyses yielded similar results to the task-specific analyses (Fig. 5), suggesting similar interpretations irrespective of task.

### Acceleration

**Fig. 7:**
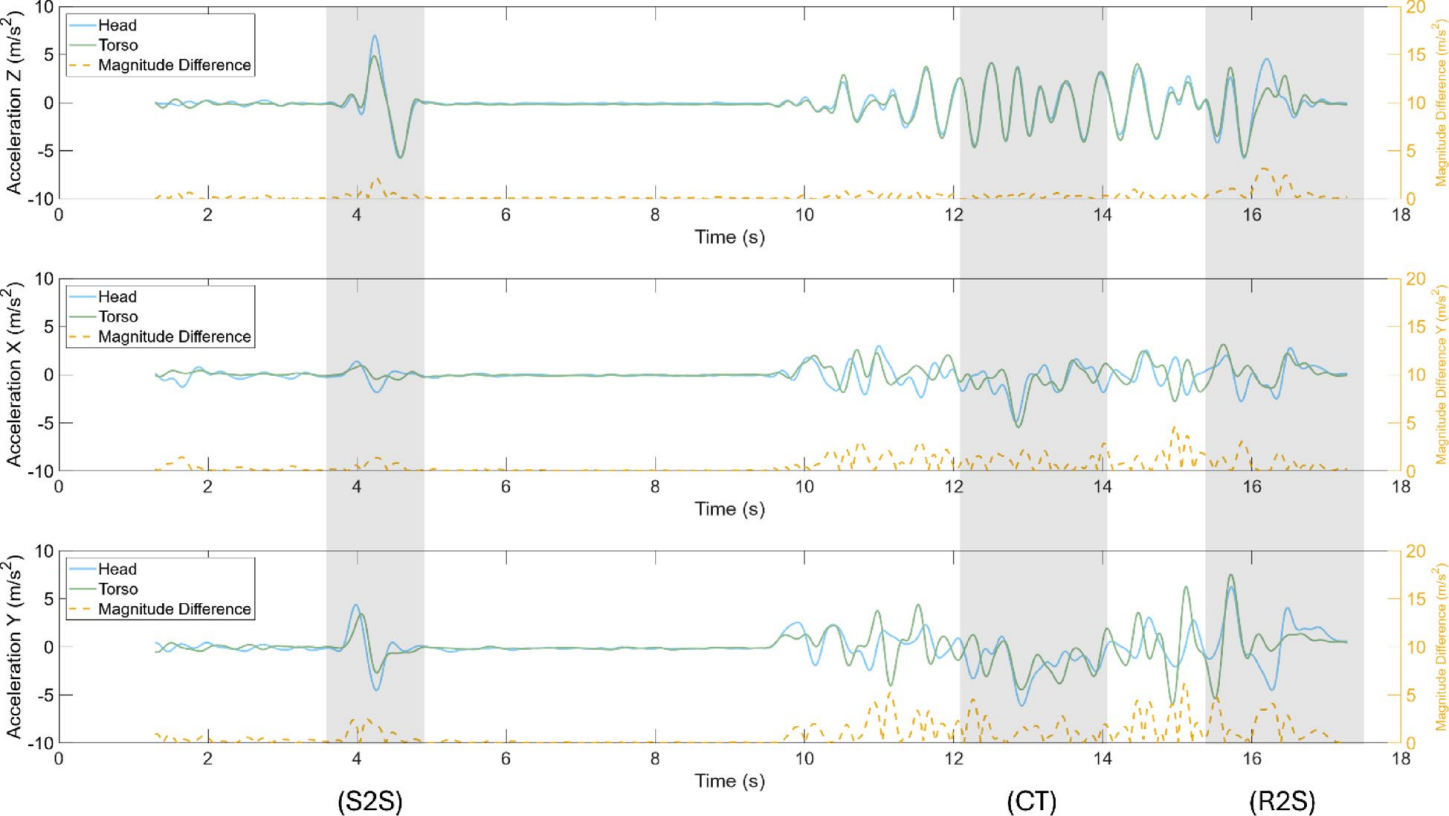
Accelerations, in the global reference frame,  of a single participant during walk and turn with the neck brace. The task phases, seated-to-stand (S2S), cone turn (CT), and return-to-seated (R2S), are shaded in grey. Left y-axis: head and trunk acceleration (m/s²); right y-axis: head–trunk difference (m/s²).

The average relative difference in acceleration along the global Z axis (vertical axis) was significantly decreased during the neck brace condition for the walk and turn task (Fig. 7), 90° turn and look, and the object translation task (Supplementary Table S3). The average relative difference along the global X axis was decreased during neck brace use for the 90° turn and look task, while an increase in the relative difference along the global Y axis was observed for the object translation task. The average coherence along the Y and Z axes increased with neck brace use for the 90° turn and acquisition task and walk and turn task, respectively (Supplementary Table S3). The recovery from fall task exhibited no significant differences between the control and neck brace condition across all the dependent measures. Task-independent analyses yielded similar results as the task-specific analyses.

### Perceptions of comfort

**Fig. 8:**
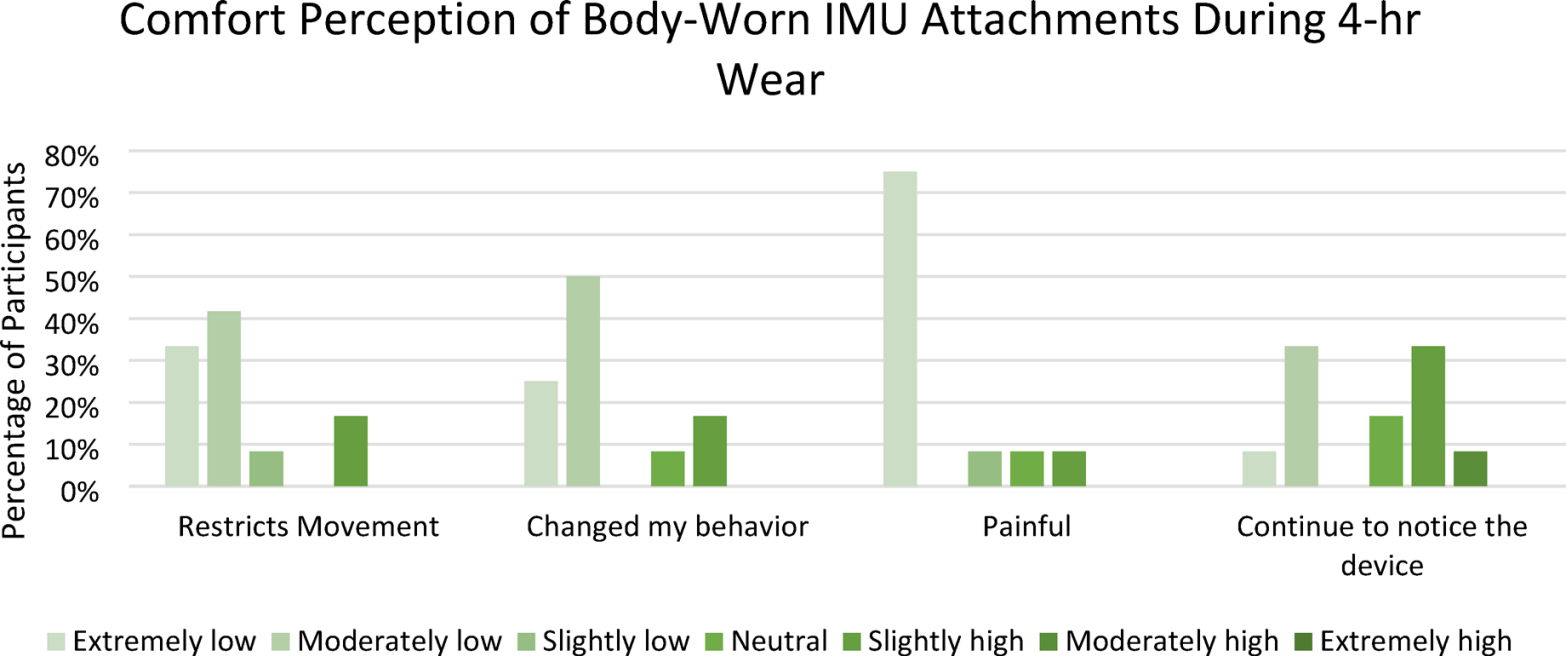
Comfort ratings for the body-worn inertial sensors during the prolonged 4-hr data collection sessions.

While 30% of participants reported higher ratings for their continued perception of the device during 4 h of wear, 75% of users reported extremely low levels of pain (Fig. 8). 83% of the participants rated low levels of perception that the body-worn devices restricted their movements or changed their behavior. Several participants noted the neoprene chest strap tended to slip down, especially while wearing shirts with low friction. One participant remarked that the balance of the brim and sensor situated at the back of the hat was ideal.

### Diagnostic accuracy of head-trunk coordination measures

**Fig. 9:**
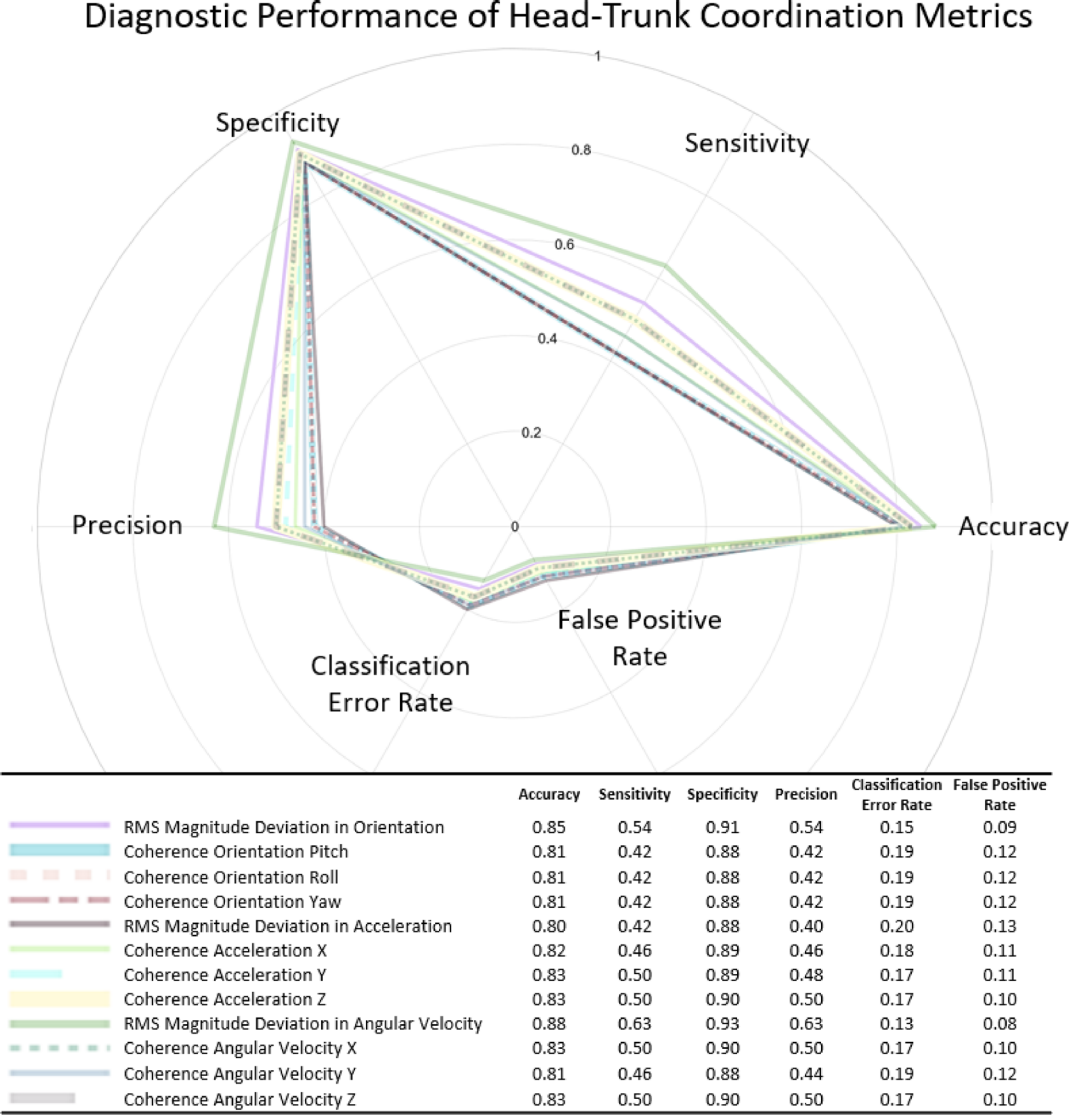
Polar plot illustrating the diagnostic performance of the selected head-trunk coordination measures. Axes represent accuracy, sensitivity, specificity, precision, classification error rate, and false positive rate, scaled from 0 to 100%.

The RMSD of the angular velocity for the head and torso was identified as the best measure of head-to-trunk coordination during neck brace use when applied to the blinded long duration dataset (Fig. 9). This measure yielded the highest accuracy (85%), specificity (93%), sensitivity (63%), and precision (63%) while also exhibiting the lowest classification error rate (13%) and false positive rate (8%). The RMSD in the magnitude orientation of the head and torso was identified as the next best identifier, followed by coherence in the angular velocity along the X and Z axes and the coherence in the acceleration along the Y and Z axes. The coherence in the angular velocity along the Y axis and the coherence in the acceleration along the X axis were better identifiers than the RMSD of acceleration and the coherence measures along pitch, roll, and yaw plans of motion, which exhibited the lowest diagnostic performance.

## Discussion

The algorithm presented in the present work builds upon previously reported IMU-based head-trunk coordination metrics by integrating them into a framework calibrated with laboratory-derived thresholds. This approach enables automated detection of head-trunk restriction in long-duration, naturalistic settings, extending prior metrics beyond controlled environments and providing a practical means to assess head-trunk coordination in real-world settings.

The results indicate that selected measures of head-trunk coordination were sensitive in detecting head-to-trunk coordination strategies as promoted by physical restriction with a neck brace. The dominant measures for improved detection accuracy were the RMSD of the angular velocity signals of the head and the torso followed by the RMSD of the magnitude orientation. The magnitude squared coherence of the angular velocity along the X and Z planes of motion also exhibited improved detection accuracy over the remaining measures. These results suggest the change in head and trunk motion was mainly driven by alterations in pitch (i.e., head tilts) and yaw (i.e., horizontal head rotation) when a head-to-trunk en bloc movement strategy was implemented. These results are further supported by the analysis of the discrete functional tasks with and without a neck brace. The relative orientation difference between the head and torso showed significant variation in pitch and yaw movements while minimal differences were observed in the roll plane of movement, despite dynamic tasks involving rolling motions such as the object translation task. Natural synchronization of the head and torso during rolling motion could be attributed to the reduced range of motion of the cervical spine in lateral flexion when compared to horizontal rotation and forward flexion and extension^25^. Horizontal rotational head movements, i.e., yaw motions, tend to be more connected to the typical actions of daily living and constitute a considerable portion of the head movements involved in maintaining balance throughout everyday activities such as directional control during locomotion^26^.

While the selected head-trunk coordination measures were successful in identifying head-to-trunk stabilization with the neck brace, the algorithm and coordination parameters also revealed instances of en bloc movement when the neck brace was not worn, as evident from the false positive rates. The false positive rate was held consistent below 13% of the total predictions across all head-trunk coordination measures with the lowest occurrence at 8%. While false positive rates concerning disease diagnosis are typically held below 10% to manage the risk of making the wrong diagnosis, there was no penalty in the present work for a slightly higher rate. Less attention was given to the false positive rate, as there were unavoidable moments where participants showed increased head-trunk coherence without the neck brace during certain activities such as monitoring a computer screen. The long duration dataset encompassed everyday activities such as walking a pet, doing household chores, and exercising. While the thresholds were helpful in identifying regions of increased head-trunk coordination, the median values for each sliding window in the long duration capture were often of lower magnitude when compared to the median values during functional task execution in the lab setting. This may be attributed to variations in subject motion when performing tasks unobserved outside of a laboratory setting or the methodology of 20-min windows, in which smaller analysis windows may better replicate the discrete functional task values with reduced timescales.

The algorithm’s advantage lies in its capability to identify head-to-trunk coordination, either voluntarily or physically influenced, over prolonged data collection, supporting potential future applications in monitoring various activities postflight for astronauts, contributing to a more comprehensive understanding of the recovery process following microgravity exposure. Currently, the assessment of crew adaptation occurs at specific intervals, such as immediately after landing, the day after, and between four to nine days later^20^. This approach offers only intermittent glimpses into sensorimotor adaptation postflight, necessitating the interpretation of recovery timelines based on minimal data points. This study can enhance additional performance and recovery metrics by gauging the percentage of recovery to preflight baseline performance to help assess crew readiness for return to daily activities. The perceptions of comfort showed low ratings for both movement restriction and pain, supporting the feasibility of body worn IMUs for several hours to provide a holistic understanding of crew adaption. However, alternate approaches using markerless motion capture are being considered for similar unobtrusive monitoring in natural settings^27^.

Concerning the findings of this work in relation to previous literature, Sreenivasa et al. (2008)^28^ investigated the relationship between head and trunk movements along various curved traversal paths and reported an increase in the maximum relative yaw of the head with respect to the torso as the angle of the turn increased from 45° to 90° to 135° and leveled off up to 180° turns. The maximum relative yaw degrees of the head with respect to the trunk for healthy participants during 90° turns were reported between 10° and 20° for unconstrained walking. The present research identified a larger magnitude for the maximum offset in the nominal control condition (median of 38.39°) for a 90° turn. This could be attributed to the distinct methodologies implemented where Sreenivasa, et al. (2008)^28^. evaluated anticipatory head movement during known walking trajectories, while the 90° turn and acquisition task in the current research assessed unanticipated or reactive turning to verbal cues, suggesting turn anticipation may influence head-trunk coordination strategies.

Khobkhun et al. (2022)^29^ quantified head-trunk coordination at various turning speeds in individuals with and without Parkinson’s disease and reported significantly smaller peak head-thorax angular separation in those with Parkinson’s across all turning speeds for 180° turns. The results also demonstrated an increase in the head-segmental separation as the speed of the turn increased, suggesting that the temporal demands of a given turning task may impact the separation of the head and trunk. In this study, the consistency of turning speed across participants was not maintained, making direct comparisons to prior research challenging due to variations in methodology and task characteristics. Nonetheless, findings indicate that participants executed rotational turns more swiftly when using a neck brace during the 90° turn and look task, displaying notably heightened torso angular velocity in the yaw plane. However, no distinctions were observed in the 90° turn and acquisition task. Significant decreases in both the maximum and average angular separation between the head and trunk were noted during the 90° turn and look task when participants used the neck brace, suggesting that despite the apparent increase in speed during turns, tight coupling of the head and torso were still maintained. Subsequent studies might explore the dynamics of turning and the impact on head-trunk coordination, especially among crew members after long-duration missions. Previous findings indicate a postflight decrease in rotational angular velocity around a cone compared to preflight conditions,^20^ making this an intriguing area for investigation.

The present study offers a controlled framework of measures and methods to assess astronauts’ postflight head movement strategies to establish further correlations with factors such as motion sickness. Although multiple measures demonstrated improved detection accuracy during extended data collection periods, it is anticipated that additional iterations of the algorithm will be conducted to refine its functionality for postflight data analysis. While the IMU attachment apparatus was deemed comfortable and unobtrusive for long duration wear, adjustments may be implemented to enhance comfort based on feedback from crewmembers. Alternatively, markerless motion capture methodology may enable unobtrusive technology, providing 3D human pose estimation from multiple synchronized 2D camera views using deep learning algorithms^27^. The developed algorithmic framework could be applied to markerless systems for real-time monitoring in analog habitats or microgravity environments; however, non-1 g thresholds would need to be established to improve accuracy in these distinct gravity conditions.

The current study constraints include the limited sample size (*n* = 12) and a cohort of healthy adults. We acknowledged that the participants may not entirely mirror the diverse backgrounds, abilities, experiences, and other characteristics found within astronaut populations, including anthropometrics and age differences. Because the algorithm functions as a flexible framework, crew-specific thresholds could be established in future applications to refine specificity for individual crew member detection. Although measures were taken to manage drift and inherent biases in the IMU data, particularly the orientation estimations, drift may have still been present especially during the long duration data captures. Wearing a neck brace might have influenced the participant’s movements because of the inherent impact associated with wearing a restrictive device. We recognize that utilizing a neck brace to physically restrict the head with respect to the torso may not provide a perfect representation of en bloc movement as it occurs in practice when voluntary restriction is applied. Subsequent studies could explore voluntary head stabilization with the torso for potential insights into the practical thresholds of movement for the present dependent measures. The sensitivity of the head-trunk coordination measures may have been influenced by the implemented protocol where discrete tasks were characterized on a time scale of seconds and minutes, while long duration data was evaluated on a larger time scale of 20 min. The selected approach aimed for efficient and practical analysis of a substantial dataset exceeding 4 h. However, altering the sliding window from 20 min to a smaller scale might have improved the sensitivity of the chosen dependent measures.

While a selective set of the significant dependent measures were evaluated in the diagnostic accuracy analysis for the long duration data, future work could evaluate alternative measures from the existing literature, such as temporal measures to support a more holistic understanding of head-trunk coordination. The present work necessitates subsequent studies to quantify crew performance using the evaluated measures during similar functional tasks. Future assessments can unveil whether the primary approach selected by crewmembers involves stabilizing the head on the torso or an alternative approach such as stabilizing the head in global space. Understanding head-trunk coordination strategies could offer insight into other influencing factors—such as motion sickness, spaceflight experience, gender, or gaze stabilization—that drive these adaptive approaches to eventually inform rehabilitation and countermeasures to promote adaptation through controlled movements. Across postflight research, crew members have exhibited response variability regarding adaptation strategies and the subsequent impact on functional performance and postural control. A bimodal response has been demonstrated across various dependent measures grouping crew members into increaser and decrease groups for example with head pitch amplitude during locomotion. Consequently, the current study’s measures are expected to reveal similar diverse adaptation strategies when applied to astronauts’ post-mission performance. Future research may also apply similar methodologies to other populations where quantifying increased head-to-trunk coordination over long duration data captures in natural settings may inform pathology-induced or age-related changes in postural control strategies.

## Supplementary Information

Below is the link to the electronic supplementary material.

Supplementary Material 1

## Data Availability

The datasets generated and/or analyzed during the current study are available from the corresponding author upon reasonable request, pending review and approval in accordance with NASA export control and data dissemination policies.
