# Supplementary material for: Development of head-trunk coordination measures for assessing sensorimotor function in laboratory and natural settings using wearable sensors
Source: Sci Rep. 2025 Dec 18;16:2508. doi: 10.1038/s41598-025-32201-9 (PMC12820101; doi:10.1038/s41598-025-32201-9)
Supplement: Supplementary file 1 — Supplementary Material 1 [file 41598_2025_32201_MOESM1_ESM.docx]

**Supplementary Methods:**

**Diagnostic Testing Accuracy Formulas**

True positives (TP) characterize correct classifications of the presence of a condition. True negatives (TN) indicate correct classification of the absence of the condition. False positives (FP) distinguish the incorrect classification of the presence of a condition. False negatives (FN) represent the incorrect classification of the absence of a condition.

1. $Sensitivity = \frac{TP}{TP+FN}$
2. $Specificity= \frac{TN}{TN+FP}$
3. $Accuracy= \frac{TP+TN}{TP+FP+TN+FN}$
4. $Precision= \frac{TP}{TN+FP}$
5. $False positive rate= \frac{FP}{TN+FP}$
6. $Classification Error rate=\frac{FP+FN}{TP+FP+TN+FN}$

**Comfort Questionnaire**

The following questions were provided in the in-lab and long duration data capture sessions. Each question was evaluated on a 7-point scale: extremely low, moderately low, slightly low, neutral, slightly high, moderately high, and extremely high.

1. The body-worn device affects the way I move. The device inhibits or restricts my movement.
2. Wearing the device makes me feel physically different. I felt strange or changed my behavior.
3. The device is painful to wear for long durations.
4. I continued to notice or feel the device on my body, and I could feel the device move throughout my activities.

**Supplementary Equations:**

1. $RMSD \omega_{{relative}_{g}}= \sqrt{\frac{1}{N} \sum_{i=1}^{N} {(|{|\omega}_{{Head}_{ig}}||-{||\omega}_{{Head}_{ig}}||)}^{2}}$

Where:

$\omega_{{relative}_{g}}$ is the relative angular velocity,

$N$ is the number of data points,

$|{|\omega}_{{Head}_{ig}}||$is the L2-norm (*Equation 2*) of the angular velocity of the head at time $i$ in the global reference frame (g),

$|{|\omega}_{{Torso}_{ig}}||$is the L2-norm of the angular velocity of the torso at time $i$ in the global reference frame (g).

1. $|{|\omega}_{{Head}_{ig}}||= \sqrt{\omega_{xg,i}^{2}+ \omega_{yg,i}^{2}+\omega_{zg,i}^{2}}$

Where:

$\omega_{x,i}$, $\omega_{y,i}$ , $\omega_{z,i}$ are angular velocities about the X, Y, and Z global axes (g) at time step $i$

$|{|\omega}_{{Head}_{ig}}||$ is the scalar angular speed (scalar magnitude) at time step $i$.

1. $C_{xy}(f) = \frac{\left| P_{xy}(f) \right|^{2}}{P_{xx}(f)P_{yy}(f)}$

Where:

$x\left( t \right)$ denotes the head angular velocity in the X global axis,

$y\left( t \right)$ denotes the torso angular velocity in the X global axis,

$P_{xy}(f)$ is the cross power spectral density of $x\left( t \right)$ and $y\left( t \right)$,

$P_{xx}(f)$, $P_{yy}(f)$ are the individual power spectral densities.

**Supplementary Tables:**

**Supplementary Table S1.** Comparative dependent measures quantifying relative differences in the head and torso orientation.

|  | **Walk and Turn Task** | | | **90° Turn and Look** | | | **90° Turn and Acquisition** | | | **Recovery from Fall** | | | **Object Translation** | | | |
| --- | --- | --- | --- | --- | --- | --- | --- | --- | --- | --- | --- | --- | --- | --- | --- | --- |
|  | *z* | *p* | *r* | *z* | *p* | *r* | *z* | *p* | *r* | *z* | *p* | *r* | *z* | *p* | *r* |  |
| Maximum Relative Difference Yaw | 2.04 | 0.042* | 0.67 | 2.04 | 0.042* | 0.50 | 2.98 | 0.001* | 0.83 | 0.08 | 0.970 | 0.00 | 3.06 | <0.001* | 1.00 |  |
| RMSD Yaw | 1.18 | 0.266 | 0.50 | 1.80 | 0.077 | 0.33 | 2.82 | 0.002* | 0.83 | 0.39 | 0.733 | 0.17 | 1.73 | 0.092 | 0.33 |  |
| Average coherence Yaw | 0.71 | 0.519 | 0.00 | -2.98 | 0.001* | -0.83 | -1.65 | 0.110 | -0.50 | 0.63 | 0.569 | 0.00 | -1.26 | 0.233 | -0.50 |  |
| Maximum Relative Difference Pitch | 2.27 | 0.021* | 0.83 | 0.16 | 0.910 | 0.17 | 1.49 | 0.151 | 0.50 | 2.75 | 0.003* | 0.67 | 2.90 | 0.001* | 0.83 |  |
| RMSD Pitch | 2.98 | 0.001 | 0.83 | -0.08 | 0.970 | 0.00 | 1.18 | 0.266 | 0.50 | 2.59 | 0.007* | 0.67 | 2.90 | 0.001* | 0.83 |  |
| Average Coherence Pitch | 0.16 | 0.910 | -0.17 | 2.12 | 0.034* | 0.50 | 0.08 | 0.970 | 0.17 | -1.49 | 0.151 | 0.50 | -1.41 | 0.176 | -0.17 |  |

Comparisons were made between the control and neck brace condition with the Wilcoxon Signed-Rank Test (z = z-score) with a level of significance of 0.05 (p ≤ 0.05) and effect sizes calculated using rank-biserial correlation (r). Significant differences indicated with an asterisk.

**Supplementary Table S2.** Comparative dependent measures quantifying relative differences in the head and torso angular velocity.

|  | Walk and Turn Task | | | 90° Turn and Look | | | 90° Turn and Acquisition | | | Recovery from Fall | | | Object Translation | | |
| --- | --- | --- | --- | --- | --- | --- | --- | --- | --- | --- | --- | --- | --- | --- | --- |
|  | *z* | *p* | *r* | *z* | *p* | *r* | *z* | *p* | *r* | *z* | *p* | *r* | *z* | *p* | *r* |
| RMSD Angular Velocity X | 2.67 | 0.005* | 0.67 | 1.88 | 0.064 | 0.50 | 1.18 | 0.266 | 0.33 | 3.06 | <0.001* | 1.00 | 3.06 | <0.001* | 1.00 |
| RMSD Angular Velocity Y | 0.94 | 0.380 | 0.17 | 1.57 | 0.129 | 0.33 | -0.16 | 0.910 | 0.00 | 1.10 | 0.301 | 0.17 | 2.98 | 0.001* | 0.83 |
| RMSD Angular Velocity Z | 3.06 | <0.001* | 1.00 | 3.06 | <0.001* | 1.00 | 3.06 | <0.001* | 1.00 | 2.90 | 0.001* | 0.83 | 3.06 | <0.001* | 1.00 |
| Average Coherence Angular Velocity X | -2.04 | 0.042 | 0.50 | -1.02 | 0.339 | 0.33 | 0.94 | 0.380 | 0.17 | -2.27 | 0.021* | 0.50 | -2.67 | 0.005* | 0.83 |
| Average Coherence Angular Velocity Y | -1.18 | 0.266 | 0.17 | 0.00 | 1.00 | 0.00 | -1.57 | 0.129 | 0.33 | -1.49 | 0.151 | 0.33 | -2.27 | 0.021* | 0.67 |
| Average Coherence Angular Velocity Z | -0.94 | 0.380 | 0.33 | -3.06 | <0.001* | 1.00 | -0.86 | 0.424 | 0.33 | -1.73 | 0.092 | 0.33 | -2.27 | 0.021* | 0.50 |

Comparisons were made between the control and neck brace condition with the Wilcoxon Signed-Rank Test (z = z-score) with a level of significance of 0.05 (p ≤ 0.05) and effect sizes calculated using rank-biserial correlation (r). Significant differences indicated with an asterisk.

**Supplementary Table S3.** Comparative dependent measures quantifying relative differences in the head and torso acceleration.

|  | Walk and Turn Task | | | 90° Turn and Look | | | 90° Turn and Acquisition | | | Recovery from Fall | | | Object Translation | | | |
| --- | --- | --- | --- | --- | --- | --- | --- | --- | --- | --- | --- | --- | --- | --- | --- | --- |
|  | *z* | *p* | *r* | *z* | *p* | *r* | *z* | *p* | *r* | *z* | *p* | *r* | *z* | *p* | *r* |  |
| RMSD Acceleration X | -0.86 | 0.424 | 0.17 | 2.43 | 0.012* | 0.50 | 1.18 | 0.266 | 0.33 | 1.26 | 0.233 | 0.33 | -0.08 | 0.970 | 0.00 |  |
| RMSD Acceleration Y | -1.18 | 0.266 | 0.17 | 1.88 | 0.064 | 0.67 | 0.78 | 0.470 | 0.17 | 0.24 | 0.850 | 0.17 | -2.27 | 0.021* | 0.50 |  |
| RMSD Acceleration Z | 2.90 | 0.001* | 0.83 | 2.82 | 0.002* | 0.83 | 1.18 | 0.266 | 0.50 | 0.00 | 1.00 | 0.17 | 2.82 | 0.002* | 0.67 |  |
| Average Coherence Acceleration X | 0.39 | 0.733 | 0.17 | -1.96 | 0.052 | 0.33 | 0.31 | 0.791 | 0.17 | -0.55 | 0.622 | 0.17 | -1.57 | 0.129 | 0.17 |  |
| Average Coherence Acceleration Y | 1.65 | 0.110 | 0.33 | 0.00 | 1.00 | 0.00 | -2.35 | 0.016* | 0.50 | 1.41 | 0.176 | 0.33 | 1.33 | 0.204 | 0.50 |  |
| Average Coherence Acceleration Z | -2.04 | 0.042* | 0.50 | -0.16 | 0.910 | 0.00 | -1.57 | 0.129 | 0.33 | -0.7 | 0.519 | 0.00 | -0.55 | 0.622 | 0.00 |  |

Comparisons were made between the control and neck brace condition with the Wilcoxon Signed-Rank Test (z = z-score) with a level of significance of 0.05 (p ≤ 0.05) and effect sizes calculated using rank-biserial correlation (r). Significant differences indicated with an asterisk.

**Supplementary Table S4.** Upper and lower thresholds of head-trunk coordination for orientation, acceleration, and angular velocity determined by performance across the discrete tasks when the neck brace was worn.

| **Orientation** | **Neck Brace Condition** | |  | **Acceleration** | **Neck Brace Condition** | |  | **Angular Velocity** | **Neck Brace**  **Condition** | |
| --- | --- | --- | --- | --- | --- | --- | --- | --- | --- | --- |
|  | *Lower*  *Threshold* | *Upper*  *Threshold* |  |  | *Lower Threshold* | *Upper Threshold* |  |  | *Lower Threshold* | *Upper Threshold* |
| Maximum Relative Difference Yaw | 19.82 | 53.86 |  | Maximum Relative Difference X | 0.97 | 1.47 |  | Maximum Relative Difference X | 14.00 | 33.85 |
| RMSD Yaw | 6.97 | 22.24 |  | Maximum Relative Difference Y | 0.93 | 1.75 |  | Maximum Relative Difference Y | 17.40 | 34.35 |
| Average coherence Yaw | 0.48 | 0.80 |  | Maximum Relative Difference Z | 0.56 | 1.56 |  | Maximum Relative Difference Z | 12.55 | 24.37 |
| Maximum Relative Difference Pitch | 18.64 | 41.26 |  | Average Coherence X | 0.22 | 0.55 |  | Average Coherence X | 0.27 | 0.64 |
| RMSD Pitch | 7.58 | 18.19 |  | Average Coherence Y | 0.26 | 0.55 |  | Average Coherence Y | 0.23 | 0.60 |
| Average Coherence Pitch | 0.34 | 0.69 |  | Average Coherence Z | 0.44 | 0.79 |  | Average Coherence Z | 0.53 | 0.83 |

Thresholds were calculated using the 25^th^ and 75^th^ percentile of task performance while using the neck brace across all discrete functional tasks.
